# An Exploratory Study of Hydrochar as a Matrix for Biotechnological Applications

**DOI:** 10.1021/acs.iecr.3c00765

**Published:** 2023-07-19

**Authors:** Alberto Gallifuoco, Alessandro Antonio Papa, Michele Passucci, Agata Spera, Luca Taglieri, Andrea Di Carlo

**Affiliations:** University of L’Aquila, Department of Industrial and Information Engineering & Economics, Via G. Gronchi, 18, 67100 L’Aquila, Italy

## Abstract

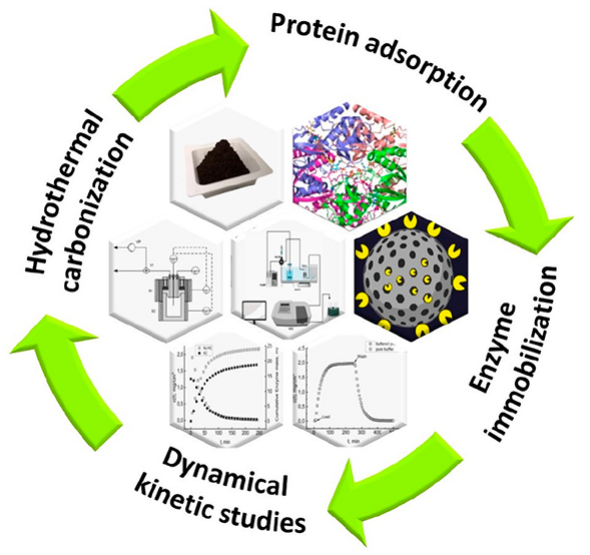

This paper explores the potentialities of hydrochar in protein separation and enzyme immobilization for non-energy biorefinery applications of hydrothermal carbonization. An innovative experimental procedure monitors soluble protein–hydrochar interactions and enzymatic reactions in a continuously stirred tank reactor. The hydrochar comes from hydrothermal carbonization of silver fir (200 °C, 30 min, 1/7 solid/water ratio) and standard activation (KOH, oven, 600 °C). Bovine serum albumin, a non-active, globular protein, was adsorbed at ≤3300 mg/g. Sip’s isotherms fitted data well (*R*^2^ = 0.99999). The immobilization used a commercial β-glucosidase, which catalyzes the hydrolysis of cellobiose to glucose, a bottleneck of the cellulose to fermentable sugar bioconversion network due to the fast enzyme deactivation. The hydrochar adsorbed ≤26 w/w% of enzyme. The heterogeneous biocatalyst operational stability was 24 times that of the soluble one. The results encourage further investigations and foreshadow process schemes coupling hydrothermal carbonization and industrial bioconversions.

## 1 Introduction

Sustaining development under increasing environmental constraints is the major challenge chemical engineers are facing. Green processes, the dominant production paradigm, should progress with integration to complete sustainability. Contemporary studies on the biorefinery concept show this trend, especially for waste,^1,2^ entrusting the non-fossil production of platform chemicals to the biorefinery breakthrough.^3^ A fully developed circular bioeconomy must consider lignocellulosic biomass in biorefinery design and optimization.^4,5^ The struggle for the price competitiveness of bio-based versus petro-based processes could only benefit from innovation and creative process optimization. The literature considers biomass hydrothermal conversions promising for the introduction of innovation into second-generation biorefinery process schemes.^6,7^ Hydrothermal carbonization (HTC) appeals to chemical engineers as the proper recipe for the multipurpose treatment of wet biomass under relatively mild conditions.^8−10^ HTC’s versatility could help overcome bottlenecks such as seasonality and variability of lignocellulosic materials, limiting the potentiality of continuous, bulk-scale conversion plants oriented to the energy and the value chains.^11^ Recent advances show the maturity of HTC to overcome the role of mere waste biomass pretreatment upstream of energy conversions. The authors share this opinion, and this study aims to proceed in this direction.

The end products of hydrothermal carbonization are a liquid phase, the process water, and an energy-densified carbonaceous solid, hydrochar (HC). The flourishing literature has reviewed the advanced, non-energy applications of hydrochars from disparate waste biomass.^12−16^ HTC of lignocellulosic materials, an environmentally benign process, yields a carbonaceous solid likely to undergo further transformation to functional matrices that can be applied to green, sustainable industrial processes of catalysis, electrical energy storage, and adsorption.^17^ Accordingly, HC recently came to the attention of researchers dealing with superperforming, hybrid materials.^14,18−20^ Innovative adsorption processes make up a vast area for HC applicability because the carbonaceous matrix allows for surface modification *in situ*, i.e., during the HTC reactions^21,22^ and postreaction.^23,24^ Gas-phase separations exploit the affinity of hydrochar for acidic compounds. The literature primarily focuses on capturing CO_2_ in the HC matrix, which is understandable considering the environmental relevance of this specific topic. HC-driven gas-phase adsorption studies usually make use of standard equipment.^25^ Recently, the use of laboratory-scale pressure-swing adsorption equipment introduced laboratory tests closer to the industrial situation,^26^ a strategy generally advisible for assessing the practical adsorptive potentiality of HC. Liquid-phase adsorption studies are the majority, especially in the removal of pollutants from wastewater.^27,28^ More information about the interactions between HC and water-soluble proteins would be advisable.^29,30^ However, the topic has potential for bulk biorefinery separation processes and innovative biocatalysis and nanoscale biomedical applications. This study enriches the specific research area and explores novel experimental approaches.

In the traditional biorefinery organization, adsorption is a specialized, high-value-added side process that would likely use the minority of HC from biomass hydrotreatment, the rest earmarked as fuel for thermal energy conversions. Bulk co-production of proteins is worth considering when biorefining macroalgae,^31^ and the unavoidable downstream separation steps could benefit from the massive use of tailor-modified HC. Also, enzyme-based biorefinery cannot do without immobilization to preserve waste-to-value bioconversion durability and biocatalyst recovery.^32^ The suitable solid support should be inexpensive, broadly available, and adequately robust to resist the stresses that arise in heterogeneous industrial bioreactors without releasing the enzyme in the liquid phase. Enzymes constitute the main operating cost of bioconversion plants, and it is imperative to ensure their reusability. Immobilization on solid matrices facilitates the biocatalyst’s downstream separation and lessens the activity decay kinetics. The literature considers physical adsorption as a more promising technique for easing implementation and reducing harmfulness toward the tertiary protein configuration. Therefore, it is always worthwhile to experimentally evaluate the performance of heterogeneous biocatalysts prepared via adsorption.^33,34^ An HC produced from the proper lignocellulosic waste could meet all of the requirements. Detailed studies of the interaction between aqueous protein solutions and carbonaceous porous materials are available.^35,36^ The authors consider that HC should deserve an analogous in-depth analysis. Large-scale applications would require an adsorbent HC prepared with simple and economic protocols, thus avoiding the sophisticated surface modifications usually adopted for high-value-added productions. Accordingly, the paper assumes simple HTC conditions,^37^ i.e., no additional chemicals in the water/biomass slurry, and a standard postreaction activation, i.e., the well-known KOH method. This study introduces a novel experimental procedure for monitoring liquid-phase adsorption under flow conditions. Therefore, the experiments use a model waste lignocellulosic biomass, silver fir sawdust, well assessed in other HTC studies and already satisfactorily activated for gas-phase adsorption tests.^26^ Raw materials of different textures, such as husks or straws, could furnish better adsorption matrices. Future research could extend this investigation over a broad range of waste biomass, searching for the optimal one. With regard to the absorbates, the tests used bovine serum albumin (BSA) and a cellulolytic enzyme, β-glucosidase. BSA has a critical role in several biotechnological conversions and applications and is a reference molecule that appears widely in protein adsorption studies at scales down to the nano dimension.^38^ Cellulases, universally acknowledged in the lignocellulosic biorefinery as central biocatalysts, suffer from thermolability, which hampers the implementation of industrial, long-lasting bioconversions. A physical immobilization based on adsorption would be a simple method for improving durability while preserving activity.

## 2 Materials and Methods

### 2.1 Experimental Section

#### 2.1.1 Preparation of Activated HC

The preparation of solid matrices was realized using a two-step procedure, with hydrothermal carbonization under standard conditions^37^ and subsequent hydrochar activation. Details of both methods are available elsewhere.^26,37^ Briefly, the waste biomass/demineralized water slurry (7/1 dry weight liquid/solid) underwent hydrothermal carbonization at 200 °C for 30 min in a 250 mL autoclave reactor. The hydrochar was separated by the process water by filtration, oven-dried (105 °C, overnight), hand-milled, and sieved (ISO 3310-1 and 2 and ASTM E11, Endecott sieves). The 106–355 μm fractions, from several identical HTC procedures, gave sufficient HC to produce in the subsequent activation step the amount of adsorbent that meets the needs of the experiments. Solid 1/2 mixtures of hydrochar and KOH (Sigma-Aldrich Corp., ACS reagent grade) were warmed under a nitrogen gas flow to 600 °C (3 °C/min), keeping the set point temperature for 1 h.^39^ After the thermochemical treatment, the material was washed to remove the inorganic salts, first with 10 wt % HCl (Sigma-Aldrich Corp., puriss grade) and then with demineralized water until a neutral pH was achieved. The samples were oven-dried at 105 °C for 24 h and stored in vials at room temperature for the adsorption and immobilization tests. Table 1 summarizes some relevant properties of HC and proteins.

**Table 1:** Some Physical Properties of Activated Hydrochar and BSA

	HC	BSA
HC average diameter (m)	2.3 × 10^–4^	–
BSA size (Å)	–	140 × 40 × 40
density (kg/m^3^)	1200	–
HC external specific surface (m^2^/g)	0.021	–
BSA hindrance surface (m^2^)	–	min, 5.7 × 10^–16^; max, 1.43 × 10^–15^
pore specific surface^26^ (m^2^/g)	281	–
average pore size (Å)	7.3	–
molecular weight (kDa)	–	66

#### 2.1.2 BSA Adsorption and Enzyme Immobilization

**Figure 1:**
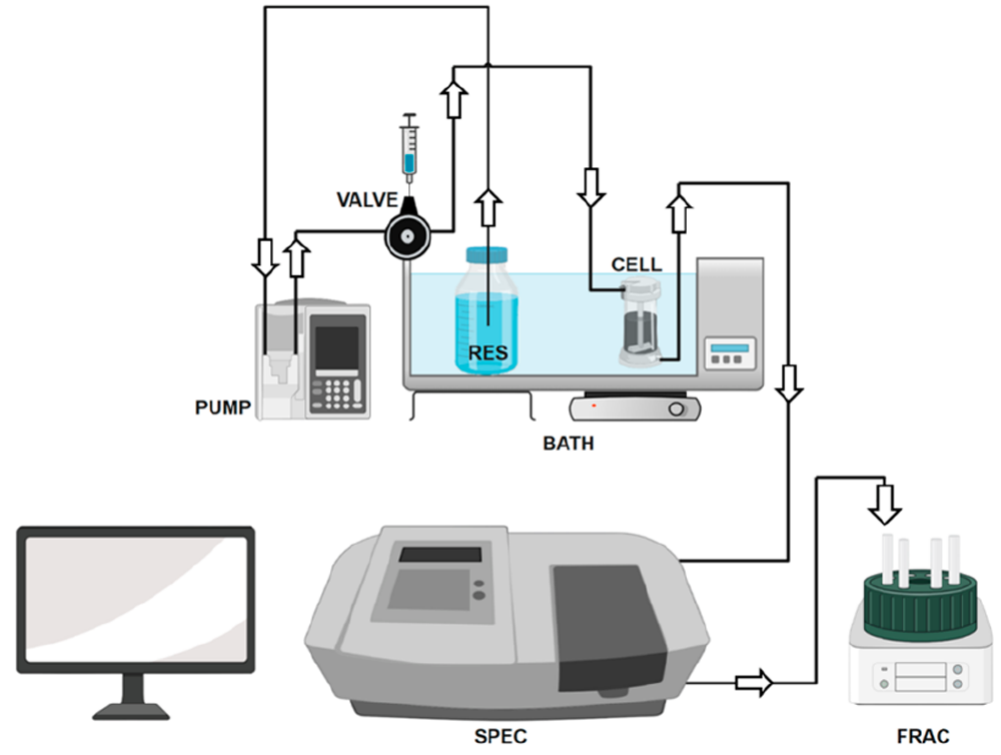
Experimental apparatus. The acronyms labeling the parts are explained in the text.

The experiments on protein–HC interactions made use of a continuous flow system set up on purpose and oriented to highlight the dynamics of the phenomenon. Figure 1 shows each device, which is designated by uppercase letters in the text.

The 1% solid suspension (120 mg of HC, unless otherwise specified) was stirred (cylindrical magnetic bar, 0.7 cm × 2 cm, 250 rpm) in a 13 mL flow cell, purposely designed and realized (CELL). A 0.5 μm microfiltration membrane (Type FH, Millipore, SA), supported by a bottom ceramic porous septum, prevented the leakage of a fine powder from the cell. A peristaltic two-channel PUMP (Gilson minipuls 3, FR) withdrew the liquid solutions from reservoirs (RES) and fed the cell to a controlled flow rate, generally set at 0.42 cm^3^/min to give a 30 min hydraulic time constant (τ). Before the adsorption tests, the HC loaded in the cell was washed for 12 h with a 20 mM buffer solution [sodium acetate/acetic acid (pH 4.8)] to remove soluble substances that could interfere with the experiments. An injection six-port VALVE (Rheodyne 3000), located upstream of the cell, allowed the inlet solution to be switched without releasing pressure or stopping the flow. The cell output crossed through a 1 mL quartz flow cuvette housed in a PerkinElmer Lambda 2 UV–vis spectrometer (SPEC) for the continuous acquisition of the optical density and, finally, was recovered in a fraction collector (Redirac 2112, LKB Bromma, SE), FRAC, to monitor the volumetric flow rate and store samples for further analyses. The interval between consecutive fractions, typically 5 min, is as short as possible to make the mixing cup concentration almost equal to the instantaneous one. The temperature was controlled at 30 °C by submersion of CELL and RES in a 20 L water thermostatic BATH. The large thermal inertia guaranteed that adsorption phenomena occurred under isothermal conditions. The negligible total volume of tubing compared to that of the CELL ensured that the instantaneous output signal tracked the system dynamics with no significant time lags. This versatile equipment configuration allows enzymes to be immobilized on HC and the operational stability of the heterogeneous biocatalyst in a unique device to be measured. This study’s experiments discuss the stirred cell’s response to inlet step concentration variations. However, feeding small volumes of a solution through the valve injection loop could extend the study to the impulse response. This latter possibility is crucial, given that it could introduce flow injection analysis as an additional tool for investigating adsorption kinetics and scaling down the apparatus could allow fast-response data acquisition to reduce the experimental time expense.

The enzyme immobilization tests used a lyophilized β-glucosidase preparation (Sigma-Aldrich, β-glucosidase from almonds). The enzyme powder, typically 24 mg, was added to the HC suspension, and the cell was kept for 24 h at 4 °C under stirring with no flow. This preliminary procedure allowed the adsorption equilibrium to be achieved while preserving the activity of the thermolabile enzyme solution. Subsequently, the temperature was increased to 30 °C, and the cell was washed with the buffered solution until no enzyme was detected in the output fractions.

#### 2.1.3 Analytical Method and Calibrations

All chemicals were ACS grade and were commercially available. The solvent was buffered water [20 mM Na acetate/acetic acid (pH 4.8)] unless otherwise specified. The concentration of BSA (Sigma-Aldrich, A2153) was measured by the 280 nm optical density compared to an eight-point calibration line that showed linearity up to 4 mg/mL. The protein content of the enzyme powder was measured by the Lowry–Hartree method against standard solutions of BSA.^34^ The dissolved β-glucosidase concentration was measured as specified for BSA. The enzyme specific activity was measured in 4 mL batch reactors containing a buffered saturating cellobiose solution and increasing enzyme concentrations. The glucose (reaction product) concentration was estimated by a d-glucose GOD-POD analytical kit (Nzytech, AK00161). The activity and stability of the HC-immobilized enzyme were measured by flowing a 20 mM cellobiose buffered solution through the cell and detecting the glucose concentration in the collected fractions for ≤48 h. The free enzyme reference tests used the same stirred cell, equipped with a UF membrane (Alfa Laval GR 81 PP) with a molecular weight cutoff, 10 kDa, that ensured the soluble enzyme retention. All of the experiments were performed in triplicate, and the standard deviation of all repeated measurements was ≤4%.

### 2.2 Modeling

The mathematical description of the experimental system dynamics discussed here is as follows. The general protein mass balance over the stirred cell is
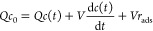
1where *Q* is the volumetric flow rate (cubic centimeters per minute), *c*_0_ and *c*(*t*) are the inlet and instantaneous liquid-phase concentrations, respectively (milligrams per cubic centimeter), *V* is the cell volume (cubic centimeters), and *r*_ads_ is the volumetric rate of adsorption on the suspended solid (milligrams per cubic centimeter per minute). Specifying the *r*_ads_ term could introduce the desired adsorption kinetic model. Setting *c*_0_ to 0 gives the mass balance during cell washout. Let IN and OUT be the cumulative mass of the absorbate entering and leaving the cell, respectively. At any time, the following relationships hold:
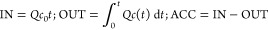
2where ACC stands for the instantaneous mass of the absorbate that accumulated in the cell in the liquid and solid phases. The integral in eq 2 is directly calculated by the spectrophotometer data acquisition software or by the assay of the fractions recovered by the collector, viz.
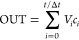
3where *Δt* is the collector time interval and *V*_*i*_ and *c*_*i*_ are the volume and concentration of the current fraction, respectively. Due to the short collection time interval adopted, the continuous and discrete calculations differ by ≤2%. ACC accounts for the total mass of the adsorbate retained in the cell, distributed between the liquid and the solid. The portion that can be attributed to the solid matrix is computed as the total minus the mass of adsorbate that accumulated in the liquid, i.e., *Vc*(*t*). Two characteristic times rule the dynamics of eqs 1– 3. The first is the cell hydraulic residence time (τ = *V*/*Q*), which is kept constant during the experimental runs. The second parameter, which could vary in progress, is the characteristic time of the adsorption process. To obtain information about the kinetics of adsorption, one should consider the cell transient response and match it with an appropriate rate equation. In any case, once the transient vanishes, the equilibrium point between the liquid concentration and mass adsorbed is detectable.

## 3 Results and Discussion

Preliminarily for the adsorption experiments, the step response of the void cell, i.e., no HC loaded, was tested. Initially containing pure buffered water, the device received a 2 mg/cm^3^ BSA solution until a steady state was reached (150 min); then, the feed switched to pure buffer. The continuous acquisition of the output signal furnished the instantaneous response of the loading and washing phases (data not shown for the sake of brevity), which followed almost perfectly (*R*^2^ > 0.9987) those of an ideal first-order system, which can be deduced by setting *r*_ads_ to 0 in eq 1. The time constant (τ) in both phases was 24.78 min.

BSA adsorption experiments proceeded with the same modalities but in the presence of HC.

**Figure 2:**
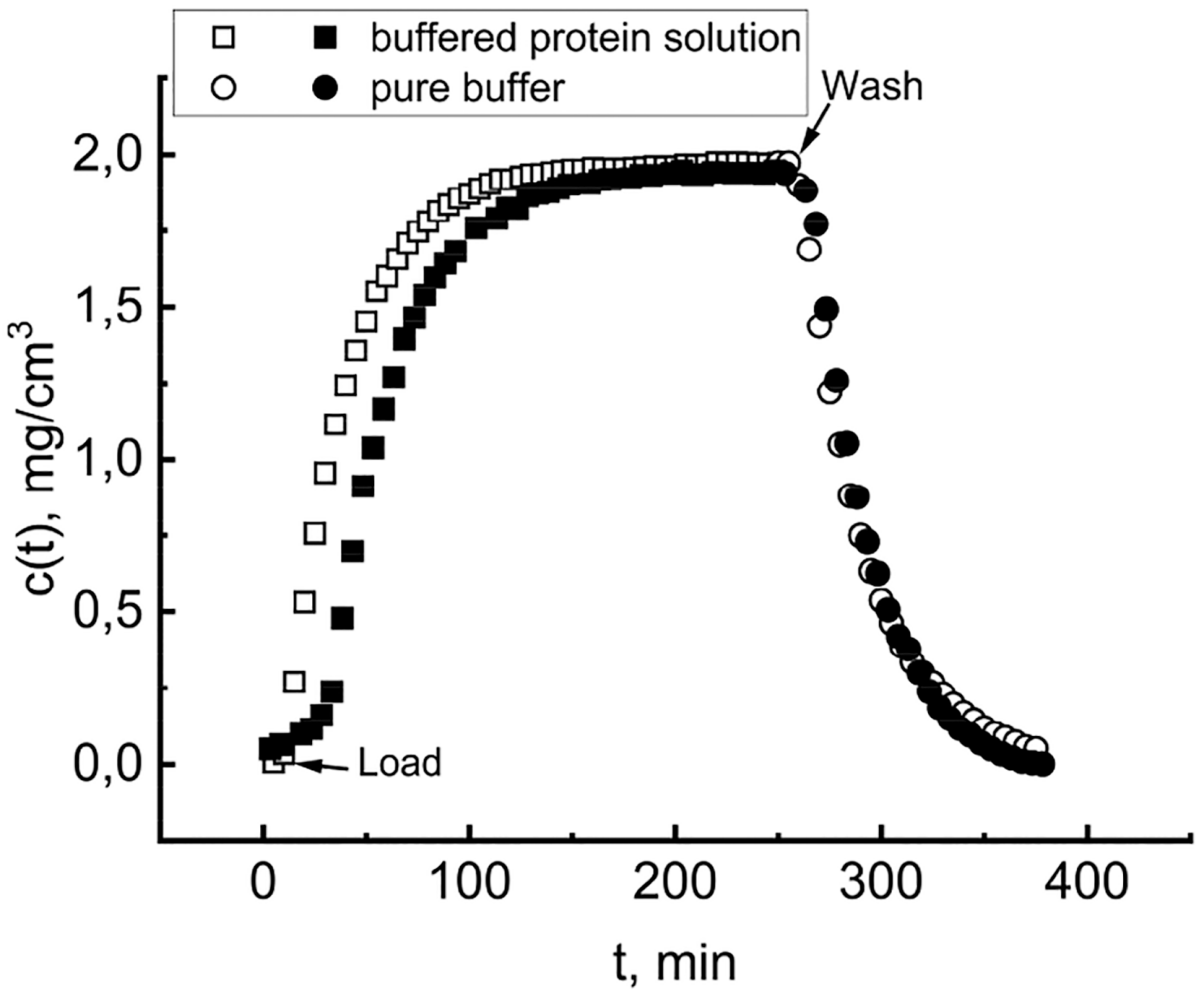
Typical dynamics of the stirred cell. The BSA concentration was determined as a function of time. Empty symbols for the void cell and filled symbols for the HC-loaded cell.

Figure 2 shows typical results, comparing the dynamics of the void cell (empty symbols) to that in the presence of HC (filled symbols). In the presence of the adsorbent, the concentration buildup during the load phase slows, while the wash phase is almost identical with and without HC. This behavior is coherent with the accumulating part of the BSA entering into the solid. As the HC approaches the saturation capacity, uptake becomes slower and eventually ceases, providing room for the residual liquid-phase accumulation. The substantial identity of the two wash phases indicates that the progressive decrease in BSA concentration does not cause the release of a significant amount of immobilized protein.

The *Y*-axis reports the BSA concentration in the exiting solution as a function of time. The cell, containing 0.12 mg of dry weight HC, was flushed overnight with pure buffer to cleanse the solid of soluble residues, which could interfere with the test. The “Load” arrow marks the entrance of the *c*_0_ = 2.3 mg/cm^3^ BSA solution and the start of recording, with a sampling frequency of one point per minute (one of five reported to avoid data crowding). The steady state, attained when *c*(*t*) equals *c*_0_, signals the equilibrium between the BSA adsorbed onto the hydrochar and that of the liquid phase in the cell. The “Wash” arrow marks when the inlet solution switches to pure buffer, and the subsequent data points track the time course of cell washout, which lasts until *c*(*t*) again is 0. Cumulative mass balances lead to quantification of the amount of BSA retained in the solid. Under the same operational conditions, the non-activated hydrochar did not adsorb any significant amount of BSA, thus indicating that the material requires a pretreatment. To reduce the costs of possible industrial applications, one should test cheaper activation methods described in the literature, e.g., based on alternative chemicals, like KHCO_3_, ZnCl_2_-based low-melting eutectic mixtures, or freeze-drying procedures.^41^ The proper strategy would be to search for a compromise between expense and performance.

A further elaboration of the data gives information about the adsorption process. According to eq 2, at a constant volumetric flow rate, IN increases linearly with time while OUT increases progressively due to the increasing outlet concentration. At the steady state, OUT becomes linear and parallel to IN, because the internal concentration buildup vanishes. Figure S2 shows how the experimental time course proceeds.

**Figure 3:**
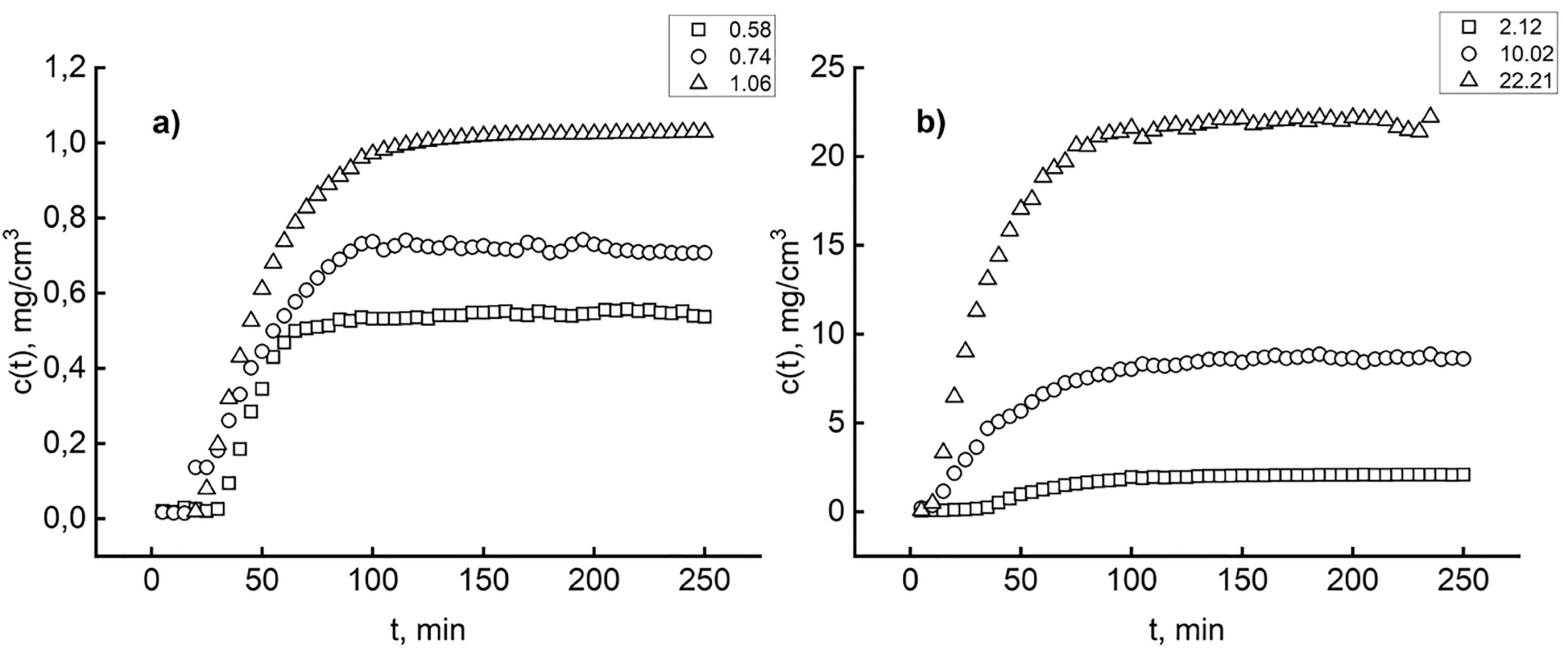
BSA concentration buildup in the outflowing solution at different inlet concentrations (milligrams per cubic centimeter): (a) low-range inlet and (b) high-range inlet.

OUT and the liquid-phase accumulation are marked with symbols, as they originate directly from experimental measurements; the other quantities appear as lines. The asymptotic tendency of OUT to arrange on a straight line parallel to IN is evident. The vertical gap between the parallel lines measures the final mass of BSA that accumulated during the load run, while the line “Total” tracks its time course. The steady-state value of the “Solid” line, 9.50 mg, is the equilibrium mass adsorbed in the presence of the *c*_0_ concentration (2.3 mg/cm^3^) in the liquid, i.e., a point of the adsorption isotherm. One could perform experiments at different inlet concentrations but load the cell with the same amount of HC to obtain the complete equilibrium curve. Figure 3 illustrates the results of this procedure.

Before each run was started, an accurate procedure guaranteed the absence of cross-contamination. The cell and the tubing were accurately cleaned by scrubbing any part with laboratory detergent to remove potentially adsorbed BSA on walls; the tubing was replaced. A fresh HC load (here, 12 mg) was inserted, and the system was flushed with pure buffer overnight. The six inlet concentrations covered a practical range from relatively low values (Figure 3a) to relatively high values (Figure 3b). In any run, the duration of 250 min ensured the cell hydraulic transient had vanished and that the steady *c*(*t*) values corresponded, within the detection limits, to the inlet ones, *c*_0_. Under these conditions, it is conceivable that the adsorption process ended so that the liquid-phase concentration is the equilibrium one, *c*_e_.

**Figure 4:**
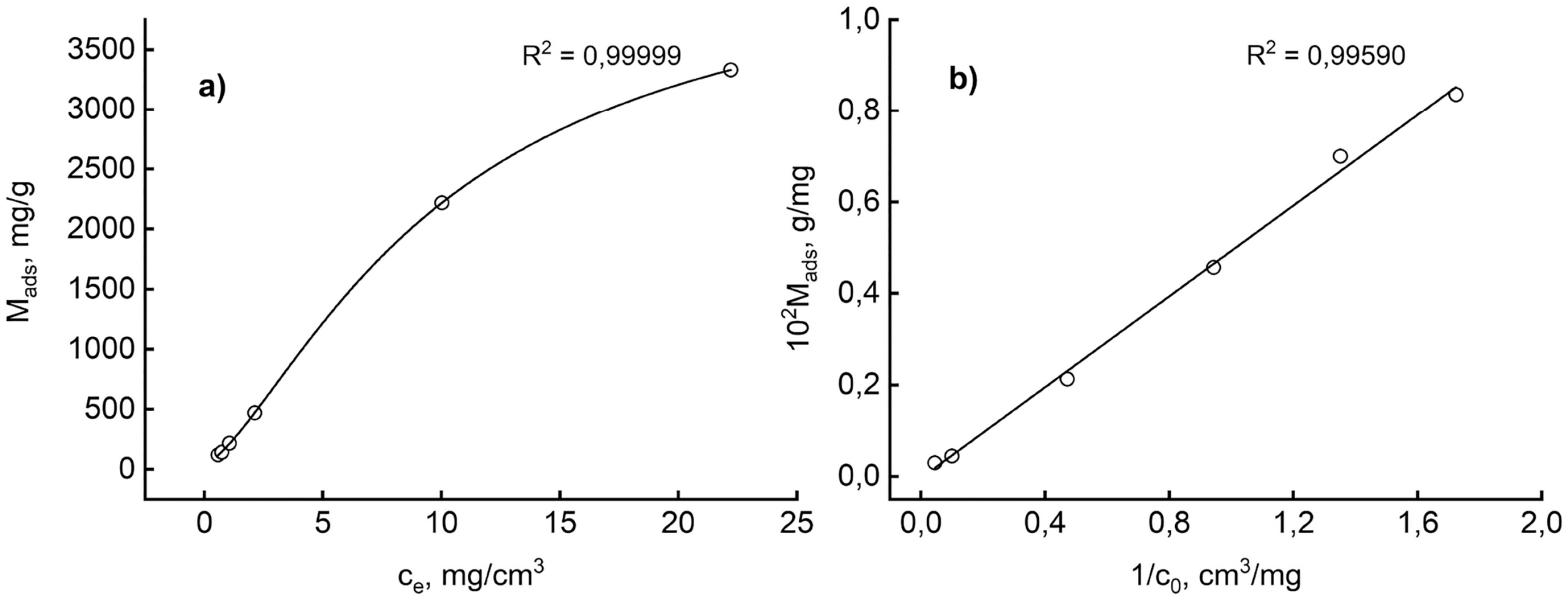
Equilibrium data fit to different isotherms: (a) Sip and (b) Langmuir.

The corresponding BSA adsorbed mass per unit HC mass, *M*_ads_, was computed by the solid-phase accumulation using the procedure described previously. Figure 4 shows that data are arranged in an orderly fashion, which confirms the validity of the experimental method expressly set up for this contribution.

Panel a displays the adsorption isotherm, while panel b represents data in the double-reciprocal plot, with the regression line connecting points. The linearity in Figure 4b should hint at a hyperbolic Langmuir-type isotherm. Despite the satisfying regression coefficient (*R*^2^ = 0.9959), this assumption is unreliable because the *Y*-axis intercept (the reciprocal of the maximum adsorption capacity) is near zero, if not negative. Data in panel a suggest the existence of an S-shaped isotherm (IUPAC type V) instead. The hypothesis should be correct if one considers the regression line reported, which corresponds to fit data with the equation
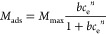
4In eq 4, termed the Sips or Langmuir–Freundlich isotherm, *M*_max_ is the saturation capacity, *b* is the affinity between the sorbate and adsorption sites, and *n* is a constant that accounts for the heterogeneity of the adsorption sites. When *n* = 1, one has the classical Langmuir isotherm; otherwise, the exponent relates to the adsorption energy distribution of the active sites on the solid surface.^42^Equation 4 explains almost perfectly the data (*R*^2^ = 0.99999) and furnishes the following: *M*_max_ = 4374 mg/g, *b* = 0.037, and *n* = 1.433. One cannot justify the satisfying quality of the regression by invoking only the higher versatility of a three-parameter model versus a two-parameter one (Langmuir isotherm). The result prompts further speculation. A type V isotherm is not unusual for mesoporous sorbents such as activated carbons, and the finding confirms that HC could be placed in this category. Proteins are quite simple sorbates in that the interaction with the active adsorption site could cause macromolecule conformational changes.^43^ The existence of a spectrum of different molecular setups could lead to cooperative adsorption phenomena, which can usually be detected by sigmoid-shaped isotherms.^44^ In the Sips context, a value of *n* of >1 confirms the cooperative binding, arguably due to an “open gate” effect of the initially adsorbed molecules on the successive bindings. On the basis of the use of binding energy distribution functions, a statistical approach can handle these complex phenomena effectively.^45^ Notably, an equation mathematically equivalent to eq 4 arises from a statistical approach to modeling hydrochar formation kinetics through cumulative frequency distributions of reaction rates.^46^ The concurrence of two such disparate phenomena to the same mathematical model is inspiring and paves the way for further conjecture, only hinted at here, but worthy of additional experimental insights beyond this exploratory study’s scope.

The estimated adsorption maximum capacity, 4374 mg/g, is relatively high, even though values of >1000 mg/g appear throughout the literature for activated carbonaceous materials comparable to HC. The novelty of the adsorbent material and the lack of an in-depth surface study, which should be examined by specialists in that area, indicate one way for future prosecution. In the framework of this contribution, a simple manipulation of the physical and geometrical properties of BSA and HC (Table 1) hints at the possible formation of a multilayer. Neither the HC particles’ outer surface nor the penetrable pores could sum up to the area required to host the protein in a single layer, neglecting the geometrical hindrance between molecules.

**Figure 5:**
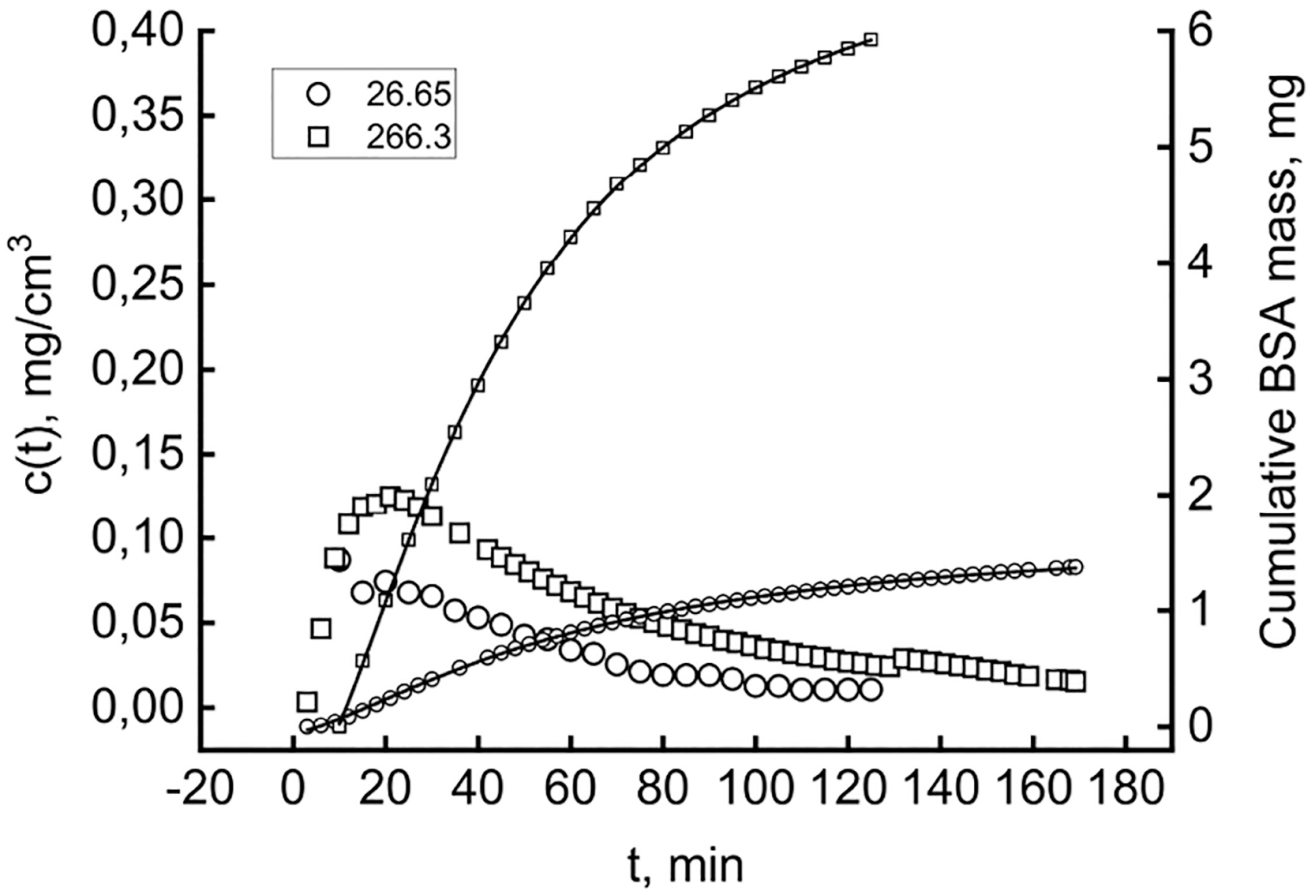
BSA desorption by a 1 M NaCl solution. The left *Y*-axis shows the time course of protein release in the outflowing stream by two differently loaded hydrochars (milligrams).

The relatively low value of parameter *b* (0.037) signals BSA’s strong affinity for HC, which would be advantageous for industrial applications such as protein removal and enzyme immobilization. At the end of the adsorption steps, the suspensions were kept in contacting pure buffer in the stirred cell without flux for ≤72 h to test the protein released from HC. The successive “Wash” steps produced an outflowing solution devoid of BSA in all cases. This result suggests very slow kinetics of protein release, if any, such as having liquid concentrations below the detectability limit. Due to the conformational and electrostatic changes, the adsorption equilibrium between dissolved proteins and active surfaces depends strongly on the liquid-phase pH and ionic strength. The industrial recovery of protein could exploit the phenomenon through the swing operational mode. Conversely, with an aim of protein removal or biocatalytic applications, the stiffness of adsorption against the electrochemical variation of the working environment would turn into an advantage. The proper sphere of HC utilization is addressed by testing the desorption behavior against stress experiments. Accordingly, the pH and ionic strength were abruptly swung, switching the inlet solution from the working buffer to the new feed. A solution buffered at pH 7.2 (20 mM Na/phosphate) provoked a negligible BSA displacement from the solid matrix to the surrounding liquid. Instead, a detectable amount of protein was transferred to the liquid using a 1 M NaCl solution in demineralized water (pH 7.0), as Figure 5 illustrates.

The left *Y*-axis refers to the time course of *c*(*t*) during the desorption from two 12 mg solid suspensions carrying different amounts of sorbate, 26.65 and 266.3 mg. In both cases, the BSA concentration peaks within the first 20 min of washing and then gradually decreases. This dynamic suggests that the characteristic time of BSA release is shorter than the hydraulic retention time (32 min) and that the relatively fast desorption slows and ceases, giving rise to cell washout. The complete kinetic study of BSA desorption would require integrating eq 1 upon inserting desorption volumetric rate equations. Recent authoritative papers^47,48^ warn of the pitfalls one could encounter and clarify how the matter deserves focused investigations. This contribution pursues short-cut objectives but is nonetheless dense with practical information, like that in Figure 5. The right *Y*-axis reports the cumulative mass of protein released during the desorption tests, as determined by eq 3. Although the phenomenon was not complete within the total run time, especially for the solid with a larger load, the tendency to set at an asymptotic value is evident. The lines connecting the corresponding time-course data points track the fitting with a typical cumulative equation, consistent with the observed *c*(*t*). The regression equation does not rest on mechanistic foundations, which can be deducted only in the presence of a more extensive array of tests.

Nevertheless, the excellent fitting (*R*^2^ > 0.9996, in both cases) makes the estimated asymptotes reliable (1.68 and 7.11 mg for 26.65 and 266.3 mg of solid, respectively). This finding demonstrates that a minor part of the total BSA adsorbed is washed out from the cell (1.45% and 0.62% for the small and large HC loads, respectively). The data suggest that, most likely, only the outer layer of adsorbed protein can undergo desorption, even after a consistent ionic strength variation. BSA is prevalently immobilized on the solid, an advantageous feature for industrial applications that aim to remove unwanted proteins from liquid effluents. Because the hydrochar sturdily immobilizes a significant portion of the protein, its reuse is unadvisable for general-purpose industrial processes of protein removal due to the overwhelming regeneration costs. On the contrary, foreshadowing HC-based heterogeneous biocatalysis, this disadvantage could turn into a virtue. For these applications, reusing a stable immobilized enzyme for repeated cycles of bioconversion is mandatory. Accordingly, the last part of this study tests the immobilization of a hydrolytic enzyme on HC.

**Figure 6:**
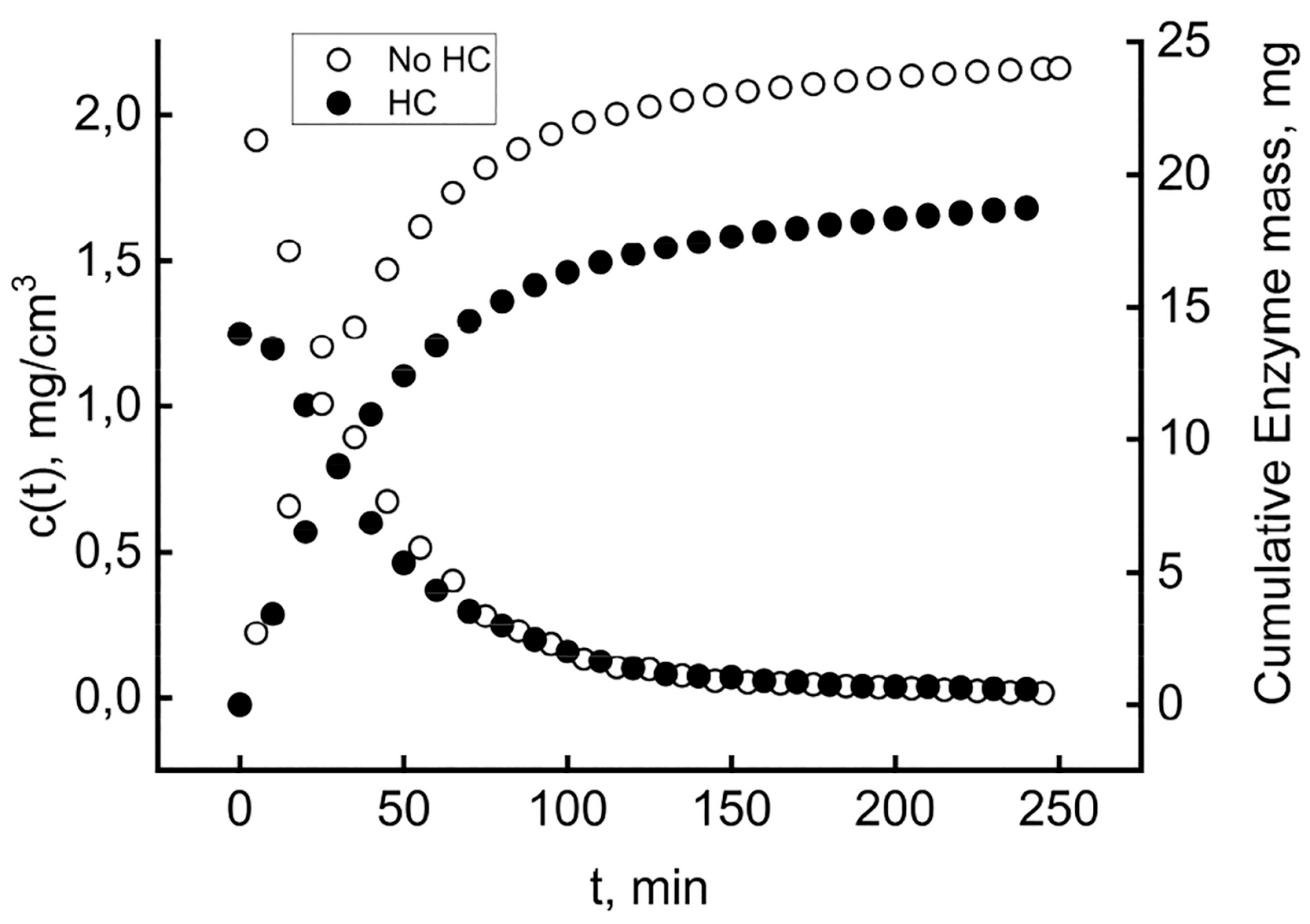
Dynamics of enzyme concentration in the cell exit stream without and with hydrochar. Left *Y*-axis for instantaneous values and right *Y*-axis for the cumulative mass.

In a typical experimental setup, after the cell containing 120 mg of HC had been washed with the pH 4.8 buffer solution, the addition of 24 mg of enzyme powder gave a liquid phase containing initially 0.0504 EU. Storage at 4 °C for 24 h without fluxing allowed the enzyme to adsorb on the solid. After an elapsed time, the gradual heating of the thermostatic bath set the temperature to the working value (30 °C), and the system underwent the “Wash” phase with the pH 4.8 buffer. The outflowing solution was recovered in the fraction collector and assayed for protein content. Runs of enzyme washout in the cell without HC allowed for the comparison. The experiments that were performed in triplicate gave substantially similar results. Figure 6 reports the typical time course recorded.

The left *Y*-axis reports the instantaneous enzyme concentration in the solution flowing out of the cell. The initial points give the liquid-phase concentration in the stirred cell at the beginning of the washout, 1.968 and 1.249 mg/cm^3^ without and with HC, respectively.

The last datum implies that 7.763 mg of protein dwells in the solid phase by the difference from the enzyme initially loaded. The washout kinetics is slightly slower in the presence of HC in the first 60 min and then becomes identical to that of the void cell. This different behavior shows that a smaller part of the adsorbed protein should be rapidly released in the surrounding liquid and that the remaining part sturdily belongs to the solid phase. To quantify the phenomenon, one could resort to eq 3 for computing the total mass of the enzyme leaving the cell. The right *Y*-axis reports these cumulative quantities. The asymptotic values give the total mass of enzyme washed out. Only 18.8 mg of the 24 mg of the enzyme was expelled in the presence of HC; the remaining mass (5.2 mg) concerns the adsorption. Accordingly, the immobilization yield, i.e., the ratio of the adsorbed enzyme to the total one, is 0.217. A prolonged washing for up to 24 h did not release detectable quantities of the enzyme in the solution flowing out of the cell. The HC strongly adsorbed the enzyme under the adopted conditions. The high affinity of the enzyme for HC ensures that the heterogeneous biocatalyst would not release the enzyme in the reactor during usage. However, this could not be a success per se, in that the strong interaction could have caused extreme variations in the protein tertiary structure. The new immobilization technique’s complete assessment requires investigation of the residual catalytic activity and stability.

To the best of our knowledge, the literature does not address the stability of enzymes immobilized onto HC or other similar carbonaceous materials from biomass treatment. However, the topic is crucial given industrial applications integrated with biorefineries where, by definition, most of the productions are long-lasting, low-value-added processes. Studies of enzyme stability may concern storage and operational conditions necessary for addressing correct industrial process development. The estimate of the operational stability is nevertheless the most important, being a laboratory test performed under conditions as close as possible to those of large-scale bioconversion.

The only reactor configuration that easily allows measurement of the operational stability is the precise one adopted for the adsorption studies. The broadness of tests with this work’s apparatus confirms its flexibility. The temperature and pH adopted for adsorptions are identical with those widely considered optimal for cellulose-hydrolyzing enzymes. Once the enzyme is immobilized, one could switch the reactor feed from a pure solution to a substrate-containing buffer solution and measure the time evolution of the product concentration flowing out of the reactor. The feed substrate concentration should be saturating; i.e., one needs to set a zero-order reaction rate to isolate the effect of the activity decay. When the typical first-order decay kinetics is included, the product mass balance is a particular form of eq 1, viz.
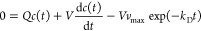
5where *v*_max_ is the maximum reaction rate and *k*_D_ is the deactivation constant. Integration of the product balance with the condition that at time zero there is no product, *c*(0) = 0, yields
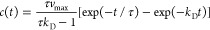
6

The dynamics displayed by eq 6 depends on the cell residence time and the deactivation characteristic time, 1/*k*_D_. Usually, the activity decay rate is much slower than the rate at which the transient hydraulics vanishes, i.e., τ*k*_D_ ≪ 1, and eq 6 simplifies to
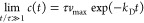
7

**Figure 7:**
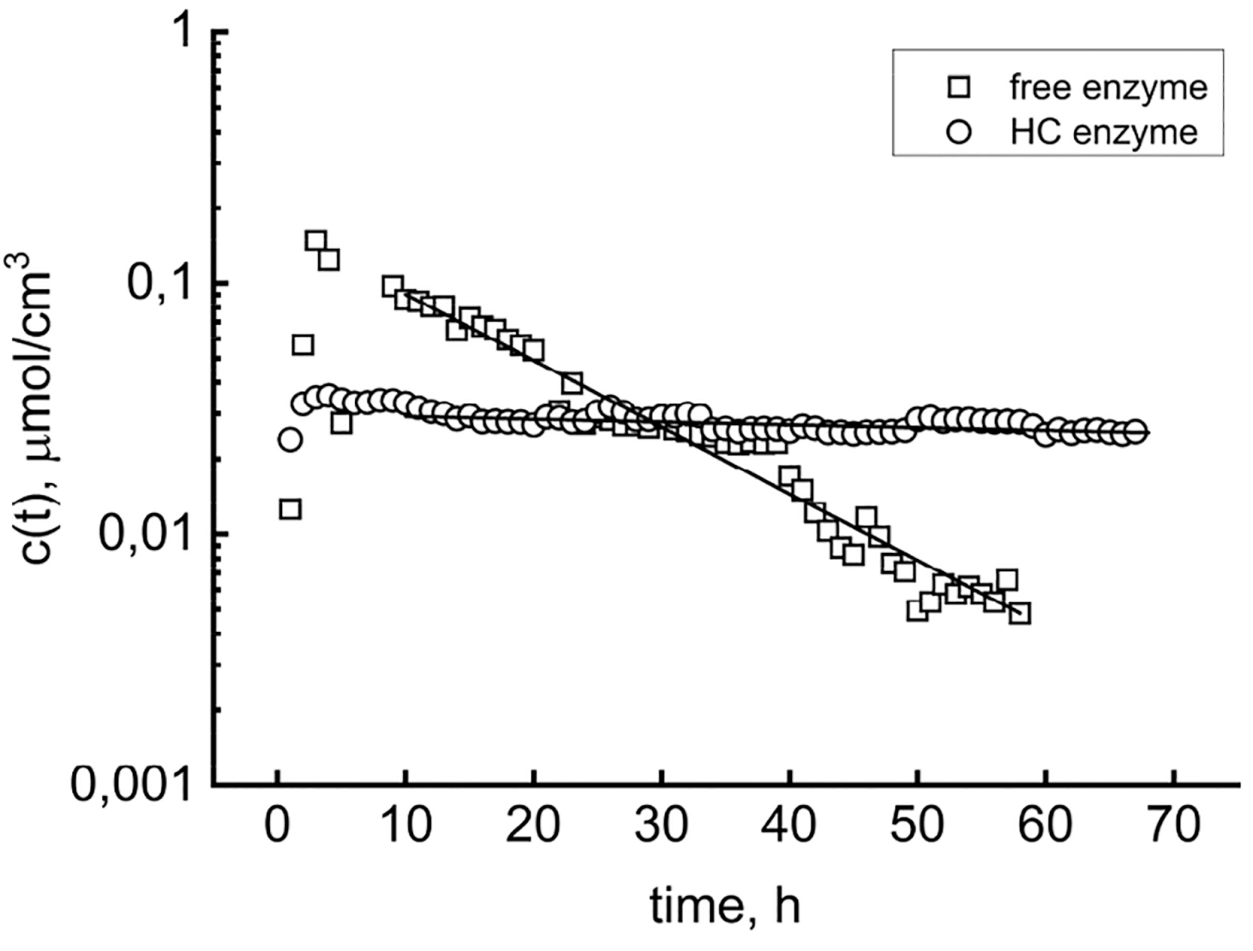
Glucose concentration as a function of reaction time during cellobiose concentration catalyzed by free and hydrochar-immobilized enzymes. Straight lines track the exponential decay correlation of the long-time data.

Consequently, in a semilog plot of product concentration versus reaction time, the experimental points should asymptotically arrange along a straight line whose slope gives the operational deactivation constant *k*_D_. Specifically performed experiments confirmed the predicted trend and provided information about the effect of HC immobilization on biocatalyst stability. The free and immobilized enzyme reactors, containing the same nominal amount of enzyme (6.36 mg), were run at 30 °C with a 20 mM cellobiose buffered solution. Figure 7 summarizes the typical results of the repeated tests, displaying the time course of the product concentration.

In both cases, the product concentration increases in the beginning due to the accumulation of the liquid-phase product. The stirred reactor behaves as a first-order system with a characteristic time τ (here, 0.5 h). Hence, one could consider the hydraulic transient to be accomplished within 3 h. Once the transient vanishes, the reactor attains a quasi-steady state, and the slow depletion of the product concentration signals the reaction rate decay. In Figure 7, the estimate of the deactivation rates uses the data points from 10 h onward. Solid lines connecting data points track the good fitting to eq 7. The slope of these straight lines gives deactivation constants of 0.061 and 0.0025 h^–1^ for the soluble and immobilized enzymes, respectively. The corresponding first-order decay half-lives are 11.4 and 277 h, respectively. Moreover, the extrapolated *Y*-axis intercepts give the maximum reaction rate and hence the specific activity of the free and immobilized biocatalyst, 7.1 × 10^–3^ and 3.3 × 10^–2^ EU/mg, respectively. The immobilized to soluble EU ratio gives an activity recovery of 0.215. The immobilization reduced the activity by a fifth but improved the stability by a factor of 24. Overall, these performances look interesting given long-lasting industrial hydrolysis processes, as evidenced by Figure 7, which shows that after 30 h, the product concentration flowing out of the HC reactor overtakes that of the soluble enzyme one. These preliminary results encourage prosecution of the investigation. Notably, the ongoing activity focuses on ascertaining the intrinsic reaction kinetics, which implies quantifying the role of possible mass transfer resistances. The literature often overlooks this central matter, which is also relevant to nonreactive protein adsorption dynamics.^49^ More in-depth investigations of external and internal mass transfer are conceivable with the new experimental apparatus, which, in the authors’ opinion, rewards its complexity higher than traditional ones, with the broader range of experiments that can be performed.

## 4 Conclusions

The hydrochar from the hydrothermal carbonization of waste lignocellulosic biomass is a good support for preparing matrices for protein adsorption, recovery, and immobilization. These non-energy applications could fit the biorefinery concept, widening the spectrum of sustainable waste-to-value chains. The innovative experimental procedure, set up here for the first time, is a useful additional tool in liquid-phase adsorption studies for investigating the equilibrium and the kinetics. Bovine serum albumin adsorbs onto the activated hydrochar according to Sip’s isotherm, and the molecule–surface interaction is strong. Using β-glucosidase as a model enzyme proved that the adsorption onto the hydrochar is a valid method for preparing heterogeneous biocatalysts essential for long-lasting green bioconversions. The immobilization highly improved the enzyme stability, while the reduction of specific activity is admissible. The result of this study could pave new ways for more in-depth investigations of protein–hydrochar interaction kinetics, bioreaction engineering, and sustainable methods for activating the hydrochar.

## Supporting Information Available

Plot of the integrated time courses, SEM images, and examples of acquisition of raw data (PDF)
