# Supplementary material for: An Exploratory Study of Hydrochar as a Matrix for Biotechnological Applications
Source: Ind Eng Chem Res. 2023 Jul 19;62(30):11805–13. doi: 10.1021/acs.iecr.3c00765 (PMC10401700; doi:10.1021/acs.iecr.3c00765)
Supplement: Supplementary file 1 — ie3c00765_si_001.pdf [file ie3c00765_si_001.pdf]

Supporting Information

An exploratory study on hydrochar as a matrix for biotechnological applications

Alberto Gallifuoco[*,1], Alessandro Antonio Papa[1], Michele Passucci[1], Agata Spera[1], Luca Taglieri[1], Andrea Di Carlo[1]

[1]University of L'Aquila - Department of Industrial and Information Engineering & Economics; Via G. Gronchi, 18 – 67100 – L'Aquila – ITALY

* Email: alberto.gallifuoco@univaq.it

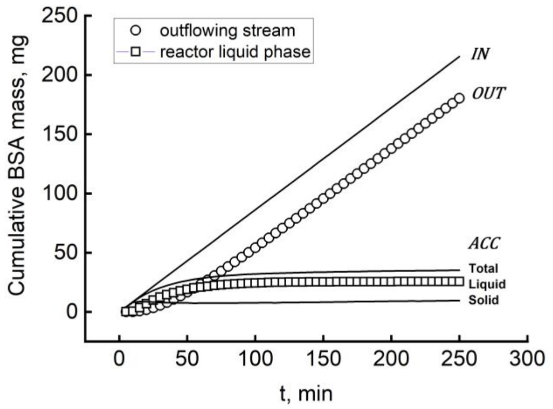

Figure S1. BSA cumulative mass distribution between the phases during the adsorption.

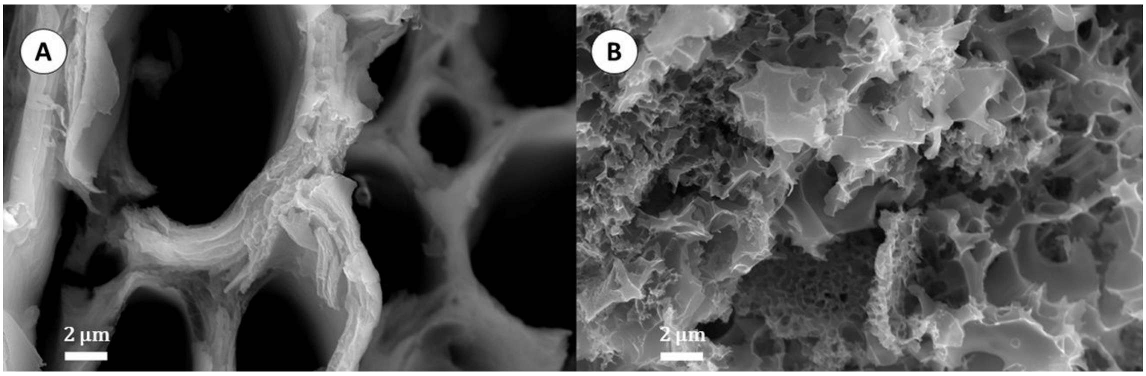

Figure S2. Typical Hydrochar SEM images (10000x) prior (A) and after (B) activation.

Loading & washing BSA 2 mg/mL no HC

BSA Initial OD	1,183	Average flowrate (loading)	25,08341 mL/h
BSA final OD.	1,157	Average flowrate (washing)	25,73129 mL/h

Sample	Time, min	Volume, mL	Flowrate, mL/h	OD (280 nm)
1	5	1,8809	22,5708	0,033
2	10	1,9619	23,5428	0,084
3	15	1,9909	23,8908	0,227
4	20	1,9677	23,6124	0,371
5	25	1,841	22,092	0,493
6	30	1,8637	22,3644	0,601
7	35	1,8715	22,458	0,693
8	40	1,963	23,556	0,765
9	45	1,9599	23,5188	0,833
10	50	2,1717	26,0604	0,885
11	55	2,0404	24,4848	0,932
12	60	2,058	24,696	0,974
13	65	2,1168	25,4016	1,004
14	70	2,0687	24,8244	1,036
15	75	2,115	25,38	1,057
16	80	2,0956	25,1472	1,077
17	85	2,246	26,952	1,092
18	90	2,0544	24,6528	1,109
19	95	2,3194	27,8328	1,115
20	100	2,1026	25,2312	1,124
21	105	2,1158	25,3896	1,135
22	110	2,1132	25,3584	1,142
23	115	2,0974	25,1688	1,148
24	120	2,1622	25,9464	1,154
25	125	2,1388	25,6656	1,156
26	130	2,1821	26,1852	1,16
27	135	2,1285	25,542	1,163
28	140	2,1412	25,6944	1,165
29	145	2,0968	25,1616	1,17
30	150	2,1276	25,5312	1,171
31	155	2,0266	24,3192	1,173
32	160	2,12	25,44	1,173
33	165	2,2654	27,1848	1,173
34	170	2,1379	25,6548	1,175
35	175	2,1986	26,3832	1,174
36	180	2,1109	25,3308	1,174
37	185	2,141	25,692	1,176
38	190	2,1773	26,1276	1,176
39	195	2,0908	25,0896	1,175
40	200	2,1068	25,2816	1,177
41	205	2,1921	26,3052	1,177
42	210	2,0332	24,3984	1,177
43	215	2,0618	24,7416	1,177
44	220	2,1288	25,5456	1,177
45	225	2,1583	25,8996	1,178
46	230	2,1393	25,6716	1,178
47	235	2,0458	24,5496	1,18
48	240	2,1255	25,506	1,18
49	245	2,1417	25,7004	1,179
50	250	2,1197	25,4364	1,179
**Start washing** 51	255	2,0444	24,5328	1,168
52	260	2,1671	26,0052	1,112
53	265	2,0446	24,5352	0,963
54	270	2,0876	25,0512	0,808
55	275	2,104	25,248	0,672
56	280	2,1213	25,4556	0,565
57	285	2,1436	25,7232	0,497
58	290	2,1323	25,5876	0,402
59	295	2,1449	25,7388	0,339
60	300	2,2006	26,4072	0,285
61	305	2,059	24,708	0,239
62	310	2,1355	25,626	0,2
63	315	2,0689	24,8268	0,177
64	320	1,9362	23,2344	0,148
65	325	1,9479	23,3748	0,122
66	330	2,1102	25,3224	0,105
67	335	2,1873	26,2476	0,087
68	340	2,0366	24,4392	0,074
69	345	2,1379	25,6548	0,063
70	350	2,1067	25,2804	0,053
71	355	1,8005	21,606	0,048
72	360	2,0912	25,0944	0,04
73	365	2,2339	26,8068	0,037
74	370	2,207	26,484	0,031
75	375	2,1805	26,166	0,024
76	380	2,0716	24,8592	0,023
77	385	2,268	27,216	0,021
78	390	2,2214	26,6568	0,018
79	395	2,2391	26,8692	0,016
80	400	2,2222	26,6664	0,014
81	405	2,1581	25,8972	0,012
82	410	2,1867	26,2404	0,013
83	415	2,203	26,436	0,013
84	420	2,2235	26,682	0,013
85	425	2,2204	26,6448	0,013
86	430	2,2239	26,6868	0,012
87	435	2,2334	26,8008	0,012
88	440	2,2181	26,6172	0,01
89	445	2,2075	26,49	0,008
90	450	2,2615	27,138	0,005
91	455	2,2288	26,7456	0,004
92	460	2,2482	26,9784	0,003
93	465	2,256	27,072	0,003
94	470	2,105	25,26	0,003
95	475	2,194	26,328	0,003
96	480	1,9841	23,8092	0,003
97	485	2,0525	24,63	0,002
98	490	2,2538	27,0456	0,002
99	495	2,1348	25,6176	0,002
100	500	2,1684	26,0208	0,001

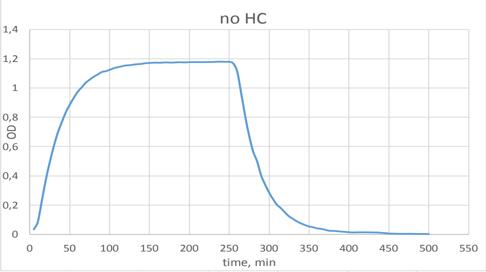

Figure S3. Typical raw data acquisition for cell BSA loading and washout.

**Loading BSA 2 mg/mL with HC**			
BSA initial OD	1,097	Average flowrate	25,85782 mL/h
BSA final OD	1,114		

Sample	Time, min	Flowrate, mL/h	OD (280 nm)
1	5	25,1808	0,004
2	10	25,8816	0,019
3	15	26,4216	0,148
4	20	26,352	0,292
5	25	26,1756	0,417
6	30	25,7688	0,527
7	35	26,8068	0,614
8	40	26,5908	0,684
9	45	26,172	0,747
10	50	25,6416	0,8
11	55	26,6424	0,855
12	60	26,0868	0,882
13	65	26,7408	0,913
14	70	26,0232	0,942
15	75	25,8432	0,963
16	80	26,2788	0,981
17	85	26,2632	0,999
18	90	25,6716	1,011
19	95	26,1732	1,023
20	100	25,644	1,031
21	105	25,7184	1,04
22	110	25,8624	1,049
23	115	25,314	1,057
24	120	26,5488	1,057
25	125	25,6164	1,062
26	130	25,7076	1,066
27	135	25,9596	1,067
28	140	25,674	1,07
29	145	26,0604	1,073
30	150	25,4892	1,075
31	155	25,6836	1,075
32	160	25,7136	1,078
33	165	26,4132	1,077
34	170	25,824	1,077
35	175	25,9524	1,077
36	180	25,9044	1,078
37	185	25,6308	1,079
38	190	25,6584	1,081
39	195	25,3272	1,08
40	200	26,0328	1,081
41	205	25,1352	1,084
42	210	25,1124	1,083
43	215	25,4424	1,084
44	220	25,5288	1,088
45	225	25,8744	1,087
46	230	24,5184	1,088
47	235	26,2128	1,087
48	240	25,8552	1,086
49	245	23,9268	1,085
50	250	26,8344	1,087

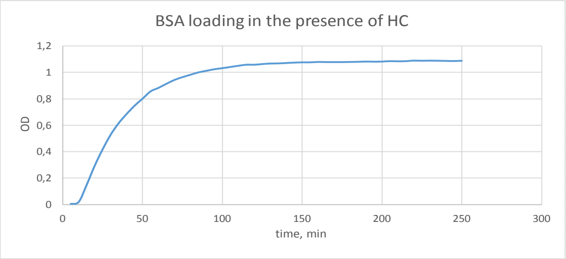

Figure S4. Typical raw data acquisition for cell BSA loading in presence of Hydrochar.

Washing BSA 2 mg/ml with HC

BSA initial OD	1,121			
BSA final OD	1,117	Average flowrate	25,22918	mL/h

Sample	Time, min	Volume, ml	Portata [mL/h]	Do (280 nm)
0	0			1,121
1	5	2,1496	25,7952	1,104
2	10	1,9599	23,5188	1,046
3	15	2,0352	24,4224	0,93
4	20	2,0231	24,2772	0,792
5	25	2,0102	24,1224	0,673
6	30	2,1103	25,3236	0,577
7	35	2,0651	24,7812	0,486
8	40	2,0334	24,4008	0,413
9	45	2,1147	25,3764	0,349
10	50	2,0736	24,8832	0,295
11	55	2,0475	24,57	0,254
12	60	2,0556	24,6672	0,213
13	65	1,6752	20,1024	0,185
14	70	2,1212	25,4544	0,165
15	75	2,0071	24,0852	0,147
16	80	2,0938	25,1256	0,127
17	85	2,108	25,296	0,108
18	90	2,1313	25,5756	0,093
19	95	2,0668	24,8016	0,08
20	100	2,0285	24,342	0,066
21	105	2,1432	25,7184	0,055
22	110	2,1552	25,8624	0,048
23	115	2,1095	25,314	0,04
24	120	2,2124	26,5488	0,031
25	125	2,1347	25,6164	0,028
26	130	2,1428	25,7136	0,022
27	135	2,1633	25,9596	0,019
28	140	2,1395	25,674	0,018
29	145	2,1717	26,0604	0,016
30	150	2,1241	25,4892	0,014
31	155	2,1403	25,6836	0,013
32	160	2,1428	25,7136	0,01
33	165	2,2011	26,4132	0,009
34	170	2,152	25,824	0,008
35	175	2,1627	25,9524	0,009
36	180	2,1587	25,9044	0,01
37	185	2,1359	25,6308	0,011
38	190	2,1382	25,6584	0,009
39	195	2,1106	25,3272	0,008
40	200	2,1694	26,0328	0,008
41	205	2,0946	25,1352	0,007
42	210	2,0927	25,1124	0,007
43	215	2,1202	25,4424	0,006
44	220	2,1274	25,5288	0,007
45	225	2,1562	25,8744	0,007
46	230	2,0432	24,5184	0,007
47	235	2,1844	26,2128	0,007
48	240	2,1546	25,8552	0,007
49	245	1,9939	23,9268	0,008
50	250	2,2362	26,8344	0,007

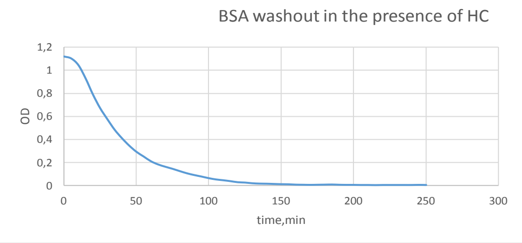

Figure S5. Typical raw data acquisition for cell BSA washout.
